# Dissecting the Molecular Mechanisms Surrounding Post-COVID-19 Syndrome and Neurological Features

**DOI:** 10.3390/ijms23084275

**Published:** 2022-04-12

**Authors:** Mohamed S. Mohamed, Anton Johansson, Jörgen Jonsson, Helgi B. Schiöth

**Affiliations:** Functional Pharmacology and Neuroscience, Department of Surgical Sciences, Uppsala University, 751 24 Uppsala, Sweden; mohamed.mohamed.8081@student.uu.se (M.S.M.); anton.johansson.6748@student.uu.se (A.J.); jorgen.jonsson@neuro.uu.se (J.J.)

**Keywords:** SARS-CoV-2, post-COVID-19, astrocytes, microglia, hypothalamus, kisspeptin, ME/CFS

## Abstract

Many of the survivors of the novel coronavirus disease (COVID-19) are suffering from persistent symptoms, causing significant morbidity and decreasing their quality of life, termed “post-COVID-19 syndrome” or “long COVID”. Understanding the mechanisms surrounding PCS is vital to developing the diagnosis, biomarkers, and possible treatments. Here, we describe the prevalence and manifestations of PCS, and similarities with previous SARS epidemics. Furthermore, we look at the molecular mechanisms behind the neurological features of PCS, where we highlight important neural mechanisms that may potentially be involved and pharmacologically targeted, such as glutamate reuptake in astrocytes, the role of NMDA receptors and transporters (EAAT2), ROS signaling, astrogliosis triggered by NF-κB signaling, KNDy neurons, and hypothalamic networks involving Kiss1 (a ligand for the G-protein-coupled receptor 54 (GPR54)), among others. We highlight the possible role of reactive gliosis following SARS-CoV-2 CNS injury, as well as the potential role of the hypothalamus network in PCS manifestations.

## 1. Introduction

Many COVID-19 survivors have reported persistent symptoms and/or the development of long-term symptoms following infection. The condition is lacking appropriate medical terminology [1], and has been referred to by multiple terms, such as post-acute COVID-19 syndrome, post-COVID-19 syndrome (PCS), “long COVID”, and post-acute sequelae of COVID-19 (PASC) [2,3,4]. Meanwhile, survivors have been described as “long haulers” [5]. For the purpose of this review, the syndrome will be referred to as post-COVID-19 syndrome (PCS).

PCS has been defined as the persistence of symptoms or the development of long-term sequelae for four weeks consequent to the onset of acute symptoms [6,7]. The prevalence of PCS has shown considerable variation between studies. A cohort study in China found that of those hospitalized during COVID-19 illness, 76% of hospital-discharged patients described at least one symptom at 6 months after symptom onset [8], and the percentage significantly decreased at 12 months, to 49% [9]. Another cohort study in the UK found that 55% of those hospitalized for COVID-19 had not fully recovered three months after infection [10]. In symptomatic COVID-19 patients, a community-based study with over half a million people in the UK estimated that around one in three experience at least one persistent symptom (for 12 weeks or more) [11]. Moreover, a prospective cohort study in Norway reported that 61% of all patients had persistent symptoms. Alarmingly, 52% of young adults (age 16–30) who were home-isolated had symptoms at 6 months, revealing that those with mild-to-moderate disease are still at risk of developing long-term persistent symptoms [12].

It has been suggested that important risk factors for the persistence of symptoms are hospitalization, increased age, and obesity [11]. Interestingly, women—and more specifically, middle-aged women—seem to have a higher risk than men of developing persistent symptoms [9,10,11,13], adding to the complex sex differences previously described for COVID-19 infections [14]. An observational cohort study has shown that those experiencing more than five symptoms in the first week are significantly more likely to develop PCS. The most predictive symptoms for the development of PCS observed in the study were fatigue, headache, dyspnea, hoarse voice, and myalgia, while anosmia (loss of smell) was the most predictive symptom in adults over 70 years [3]. A longitudinal multiomics study revealed that type 2 diabetes, SARS-CoV-2, RNAemia, EBV viremia, and autoantibodies at the time of COVID-19 diagnosis are risk factors for PCS [15]. On the other hand, a recent prospective cohort study showed that persistent symptoms are independent of the severity of initial illness [12], and a similar Mediterranean cohort study with structured assessment interventions concluded that neither the initial severity of COVID-19 illness nor age can act as an independent predictor of the development of PCS [16].

While there is a broad consensus on the high prevalence of PCS significantly affecting the quality of life of patients, little is known regarding the mechanisms behind the manifestations. The significant heterogeneity of available studies, coupled with their contrasting methodologies, has made it more difficult to reach a clear consensus regarding the mechanisms [17]. In this review, we look at the characterization of PCS, previous post-SARS syndromes, and their mechanisms. We explore in particular the potential role of reactive gliosis in the neurological manifestations of PCS, as well as the potential involvement of the kisspeptin–hypothalamus network.

## 2. Post-COVID-19 Syndrome Characterization

The current literature on PCS describes a wide range of symptoms and multisystem involvement [4,8,18] (Table 1). It has been demonstrated that after 30 days of COVID-19 illness, patients have a higher risk of death and greater need for medical resources [4]. Notably, a relatively high frequency of pain, antihypertensive, antidepressant, and anxiolytic medications, as well as multiorgan laboratory abnormalities, have been reported [4]. The respiratory system is the most disrupted system during SARS-CoV-2 infection, and the detection of persistent and/or new pulmonary symptoms is critical for the characterization of PCS. Likewise, radiological pulmonary abnormalities were detected in 71% of COVID-19 survivors 3 months after recovery [19], in roughly 50% at 6 months [8], and persisted at 12 months [9]. Pulmonary function is also impaired, with altered diffusion capacity in 39% of patients, as well as restrictive (15%) and obstructive (7%) patterns reported [20]. The most reported symptom of the respiratory sequelae of PCS is dyspnea, in 40–60% of long haulers [21,22]. In addition, cardiac manifestations have also been described among survivors, with roughly 67% reporting palpitations and 29–53% experiencing chest pain [18,23].

PCS is a heterogeneous condition, and is likely to involve multifactorial processes. Nonetheless, PCS studies with higher numbers of participants have shown consistent clusters of symptoms. An international observational cohort study, performed by a patient-led research collaborative for long-COVID survivors, has shown that fatigue, post-exertional malaise, and cognitive dysfunction are among the most common symptoms described by these patients [18]. This is consistent with a previous large cohort study in China that showed that 6 months post-infection, most COVID-19 survivors struggled with fatigue, anxiety, and depression [8]. Furthermore, a recent longitudinal follow-up study in Germany has also reported similarly alarming findings. The study showed that the neurocognitive symptoms can persist up to one year after symptom onset, significantly affecting the quality of life in patients [24]. The persistent fatigue in PCS is independent of initial infection severity or laboratory markers of inflammation [25]. Alarmingly, the most common PCS symptoms (dyspnea, anxiety, and depression) are reported more frequently at 12 months than at 6 months after COVID-19 illness [9].

The immunological mechanisms behind the persistence of SARS-CoV-2 symptoms have been broadly discussed, and include immune exhaustion leading to chronic inflammation, autoimmunity, and imbalances of the renin–angiotensin system (RAS) [26]. Neurocognitive symptoms of PCS show an association with high antinuclear antibodies (ANAs), supporting the claim for autoimmune etiology [24]. In addition, mast cell activation syndrome (MCAS) has also been linked to PCS [27]. Furthermore, a longitudinal study investigating the long-term SARS-CoV-2-specific immune responses using saliva samples found no association between hat viral shedding, PCS symptoms, and immune responses, hinting that if persistent viral stimulation is associated with PCS, it is likely to be in deeper tissues. The study showed lower frequency of degranulation of virus-specific CD8^+^ T cells in the PCS population [28].

### 2.1. Previous SARS Outbreaks and Long-Term Complications

Long-term studies on the post-viral sequelae of previous severe acute respiratory syndrome (SARS) outbreaks can aide in understanding PCS. The available literature indicates persistent symptoms in a high proportion of patients. For instance, roughly 16% of SARS survivors have pulmonary impairment and decreased exercise capacity at 6 months after disease onset [29]. At one-year post-illness, 28% of survivors have radiological abnormalities, and 24% show impairment in diffusion capacity assessment (Dlco) [30]. Abnormal chest radiographs are also reported following Middle Eastern respiratory syndrome (MERS) outbreaks [31]. In survivors with abnormal findings, 33% had lung fibrosis [31]. The respiratory sequelae have been shown to be correlated with severe pneumonia at disease onset [32]. Moreover, the respiratory impairment persisted for two years among SARS-CoV-2 survivors, with impaired Dlco and exercise capacity reported [33]

In addition to the long-term respiratory impact, neurological symptoms have also been reported. A long-term follow-up study after the SARS outbreak in 2003 found that 40% of respondents reported chronic fatigue and 27% met the criteria for chronic fatigue syndrome (ME/CFS) diagnosis, while mental disorders were reported by 40% of respondents [34]. Another case–control study described chronic post-SARS symptoms of persistent fatigue, chronic pain, and depression [35]. Likewise, survivors following MERS illness reported chronic fatigue and depression at 12 months after disease onset [36]. Depression and post-traumatic stress disorder (PTSD) requiring psychiatric management were reported in about 18% of SARS survivors at 24 months post-infection [33].

Overall, previous studies on SARS outbreaks are limited by small sample sizes, but demonstrate similarities to PCS. The neurological manifestations observed in PCS and post-SARS are not unique to coronaviruses. While largely overlooked, neurological sequelae have been reported in several neurotropic pathogens, such as enteroviruses, chikungunya virus, West Nile virus, Zika virus, and influenza viruses. Additionally, conditions such as post-Lyme syndrome reveal striking similarities to PCS manifestations [37], and both syndromes—as well as chronic fatigue syndrome (ME/CFS)—share similar disrupted immunological and metabolic pathways, such as MAPK dysregulation [38].

### 2.2. Reactive Gliosis and Excitotoxicity in the Wake of SARS-CoV-2 CNS Injury

**Figure 1:**
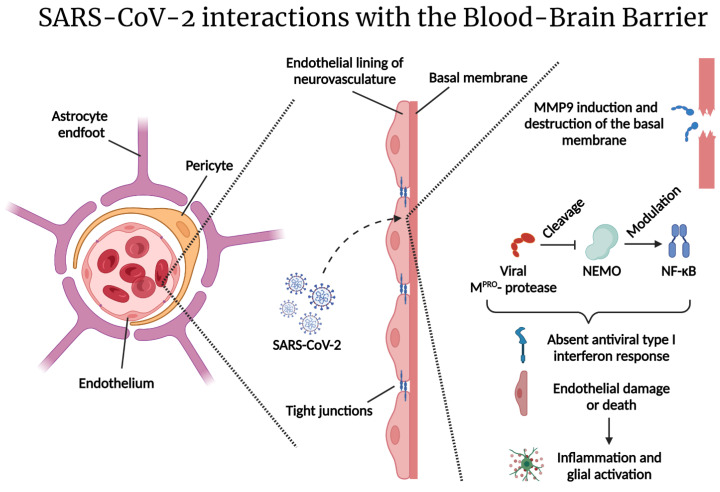
SARS-CoV-2 interactions with the blood–brain barrier: The blood–brain barrier is a semi-permeable barrier composed of microvascular endothelial cells, pericytes, and astrocyte end-feet. Tight junctions restrict paracellular passage of substances and pathogens. SARS-CoV-2 can pass via the transcellular pathway and disrupt the basal membrane. SARS-CoV-2 increases matrix metalloprotease (MMP) expression, leading to the destruction of collagen IV—a major component of the basal membrane [43]. The SARS-CoV-2 protease M^pro^ may cleave NEMO—a protein that regulates the transcription of antiviral type I interferons [42]. The consequences of these processes lead to endothelial damage, inflammation and, subsequently, glial activation. Figure was created with the BioRender software.

A large percentage of COVID-19 patients’ clinical presentations reveal signs of neurological involvement. Up to 80% of hospitalized patients show neurological manifestations [39]. These manifestations may evolve due to the direct viral invasion of the CNS and/or due to the hyperinflammatory state caused by the infection [40]. Direct SARS-CoV-2 brain infection remains a matter of debate. CNS injury is a well-recognized feature of COVID-19 infection, and SARS-CoV-2 has been described as a neurotropic virus. SARS-CoV-2 utilizes ACE2 as the receptor for host entry. Some studies have shown ACE2 expression in endothelial cells, pericytes, and astrocytes, suggesting a hematogenous route of neuroinvasion compromising the blood–brain barrier (BBB) and/or the blood–cerebrospinal fluid (CSF) barrier [41]. Recently, it has been shown that the SARS-CoV-2 protease (M^pro^) cleaves the nuclear factor kappa B (NF-κB) essential modulator (NEMO), inducing endothelial cell death, BBB disruption, and neuroinflammation [42]. SARS-CoV-2 has also been shown to cross through the microvascular endothelial cells via the transcellular pathway, without disrupting the tight junctions [43]. Subsequently, leakage in the barrier can lead to the entry of pro-inflammatory cytokines, promoting a neuroinflammatory state (Figure 1) [41]. On the other hand, some studies have suggested that the virus might enter the CNS from the periphery. For instance, SARS-CoV-2 may utilize the olfactory epithelium to gain access through the olfactory [44] or the terminal cranial nerves [45], providing the virus with a direct route to targets in the brain. Alternatively, SARS-CoV-2-derived structural protein (S1) subunits have been shown to act as pathogen-associated molecular patterns (PAMPs), initiating neuroinflammatory processes leading to the activation of astrocytes and microglia in vitro [46].

Indeed, emerging evidence has shown that SARS-CoV-2 can infect astrocytes after reaching the brain, and can induce neuropathological changes in patients [47]. Biomarkers of CNS injury are elevated in both CSF [48] and plasma samples [49] of COVID-19 patients. In both studies, GFAP—a marker for activated astrocytes—was detected. Moreover, postmortem analysis of COVID-19 patients displayed hypertrophic astrocytes and activated microglia [50]. Astrocytes can thus play a pivotal role in the neuropathology of COVID-19, being involved in the virus’ CNS spread, immune responses, and neurons’ function [51].

**Figure 2:**
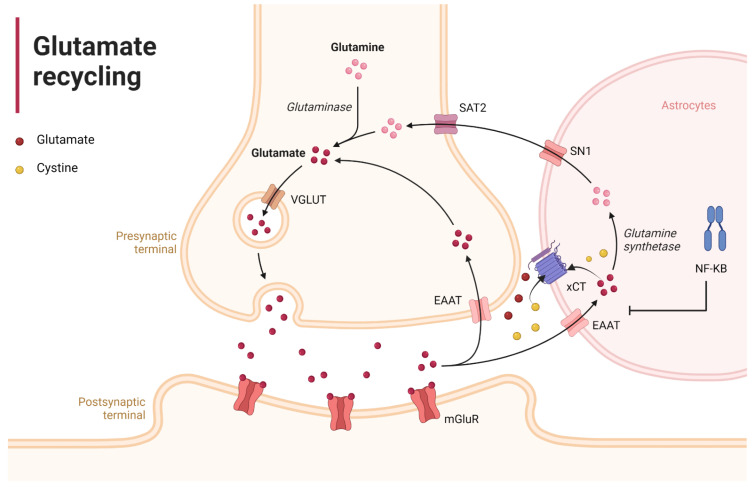
Glutamate recycling: Glutamate (red-filled circles) is quickly cleared from the extracellular space. The EAAT2 in astrocytes is responsible for 90% of the reuptake, and its expression is regulated by NF-κB [54]. Glutamate is first converted by glutamine synthetase and then transported back to the extracellular space by the System N (SN1) glutamine transporter. Glutamine is then transported into neurons by the System A transporter (SAT2) and, subsequently, converted back to glutamate by glutaminase to complete the cycle [56]. In addition, astrocytes can release glutamate through the cystine/glutamate antiporter (xCT). This antiporter takes up extracellular cystine and releases intracellular glutamate [57]. Figure was created with the BioRender software.

Under normal physiological conditions, astrocytes play an important role in synaptic regulation. For instance, net synthesis of the most essential neurotransmitters—glutamate and GABA—is under astrocytic control [52]. Moreover, glutamatergic (Figure 2) as well as GABAergic and glycinergic synapses are heavily dependent on astrocyte reuptake and recycling of neurotransmitters, and astrocytes contribute to both short-term and long-term synaptic plasticity [53]. Among these, glutamatergic synapses are well known for their plasticity, and are especially vulnerable to undergoing permanent changes via misguided long-term potentiation (LTP) [53]. Glutamate is the most abundant neurotransmitter in the brain, and influences several bodily functions and sensations [53,54]. The excitatory amino acid transporter 2 (EAAT2) in astrocytes is responsible for 90% of the glutamate uptake from the extracellular space, and its expression is regulated by NF-κB [55]. Upon uptake, glutamate is first converted to glutamine by the glutamine synthetase enzyme in the astrocytes. Then, glutamine can be released into the extracellular space to be picked up and converted back to glutamate by the neurons. This cycle has been shown to play a vital role in maintaining excitatory neurotransmission [56]. In addition, astrocytes can release glutamate through the cystine/glutamate antiporter (xCT). This antiporter takes up extracellular cystine by releasing intracellular glutamate at a ratio of 1:1, and is mainly expressed astrocytes. Cystine uptake through the xCT is important for glutathione production, neuroprotection from oxidative stress, and modulation of metabotropic glutamate receptor (mGlur) signaling via the xCT-mediated glutamate release [57,58].

Under pathological conditions, in response to CNS injury and neuroinflammation, astrocytes become reactive. Reactive astrogliosis can induce NF-κB activation [59], leading to EAAT2 downregulation and, subsequently, an increase in extracellular glutamate, contributing to neurocognitive symptoms [60] and altered cardiorespiratory function [61]. Furthermore, glutamate reuptake in astrocytes is essential for maintaining normal xCT activity. Reduced glutathione levels can lead to an increase in reactive oxygen species (ROS) production [62]. Moreover, altered xCT function has been shown to induce hyperexcitability, overactivation of extrasynaptic NMDA receptors, and ischemic damage [63,64]. While reactive astrogliosis is considered a defensive mechanism, increasing neuroprotection and the formation of astroglial scars to isolate lesions, reactive astrocytes contribute to neurodegenerative processes and neuropsychiatric disorders [60]—conditions that have been described in most PCS cases [8,18,24].

Interestingly, recent studies have shown that the microglia play an essential role in initiating the process of reactive astrogliosis by triggering the NF-κB signaling pathway [65]. Microglia can also play a direct role in PCS. It has been hypothesized that after neuroinflammation, hyperactivation of P2X7 receptors (an ATP-gated ion channel expressed mainly in the microglia, but also in astrocytes) by ATP released from distressed cells can lead to subsequent glutamate release and ROS formation, contributing to neuropsychiatric (NP) manifestations [66]. Furthermore, ATP can act as a damage-associated molecular pattern (DAMP), which activates NF-κB in the microglia, upregulating pro-inflammatory cytokines and activation of the NLRP3 inflammasome [67]. Higher concentrations of extracellular ATP can also activate NF-κB in astrocytes through IL-1β activation [68]. Finally, the microglia can also contribute to NP symptoms in PCS, due to their role in monoamine metabolism. The microglial release of pro-inflammatory cytokines activates indoleamine 2,3-dioxygenase (IDO)—an enzyme that metabolizes tryptophan to kynurenine, reducing its availability for serotonin synthesis. The activated microglia then convert kynurenine into quinolinic acid—an NMDA receptor agonist—contributing to more glutamate release [69].

The most common symptoms in PCS seem to overlap with ME/CFS, and roughly half of PCS patients at 6 months fulfilled the criteria of ME/CFS diagnosis [70]. Moreover, shared molecular signatures have been reported between the conditions [38]. ME/CFS is a chronic condition with unknown etiology. However, the activation of astrocytes and microglia seems to play a role in its development [71,72]. Chronic pain observed in both conditions can be attributed to central sensitization (an increased progressive responsiveness to neuronal signaling within the CNS) [73]. Central sensitization has been shown to predict fatigue independently of musculoskeletal pain [74], and is linked to ME/CFS [75,76]. Intriguingly, astrocytes have been shown to play a role in central sensitization by regulating synaptic plasticity through EAAT2 expression [77]. Hence, the abovementioned processes of neuronal–glial and glial–glial interactions are considered critical in the development of chronic pain, and chronic pain has been described as a gliopathy [78,79].

**Figure 3:**
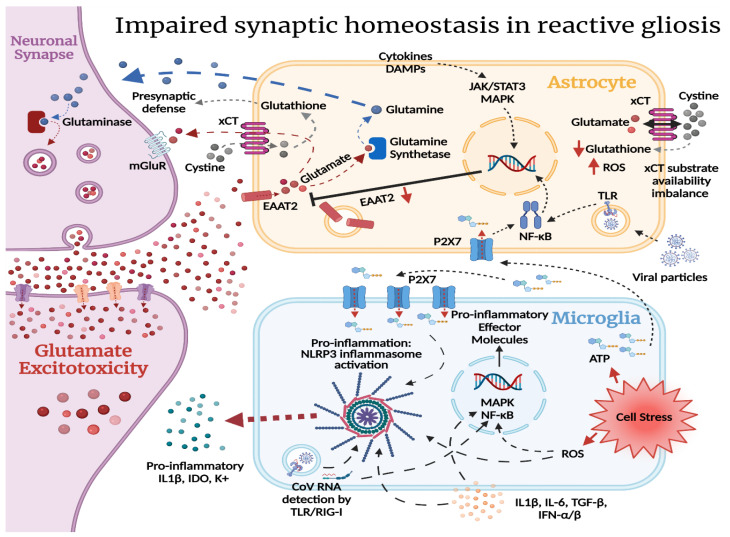
Glial activation and excitotoxicity following COVID-19 illness: In glutamatergic synapses, glutamate is transported into astrocytes by the EAAT2 transporter. It can be transported back to the pre-synapse in the form of glutamine, after being converted by the astrocytic glutamine synthetase. Glutaminase in the pre-synapse converts glutamine into glutamate for vesicular storage [55]. Glutamate also fuels the astrocytic xCT antiporter. Glutamate released from the astrocytes regulates synaptic activity through extrasynaptic inhibitory mGluR receptors. The antiporter also facilitates cystine’s entry into the astrocyte—a molecule important for glutathione synthesis [56]. Glutathione is the first line of antioxidative defense of the cell, and both cystine and glutathione are transported to the pre-synapse for the same reasons. During COVID-19 infection, both direct viral invasion and neuroinflammation are potential contributing factors to reactive gliosis. In astrocytes, NF-κB downregulates the EAAT2 glutamate transporter [59]. Reduced astrocytic glutamate uptake from the synaptic cleft promotes glutamatergic signaling in the synapse, potentially leading to glutamate excitotoxicity. Lowered amounts of intracellular glutamate in the astrocytes create a substrate availability imbalance for the xCT antiporter, wherein less cystine is transported into the astrocyte. This negatively impacts glutathione synthesis, increasing the susceptibility of astrocytes and the pre-synapse to oxidative damage in a neuroinflammatory environment [56]. In microglia, ATP can act as a damage-associated molecular pattern (DAMP) and activate NF-kB, leading to activation of the NLRP3 inflammasome. DAMPs, PAMPs, and cytokines signal the cells to enter a reactive state. ATP released from distressed cells can lead to hyperactivation of the P2X7 receptor contributing to glutamate release and ROS formation. Higher levels of extracellular ATP can also activate P2X7 in astrocytes, adding to the increased glutamate in the extracellular space. The pro-inflammatory signals may activate signaling pathways such as JAK/STAT3, MAPK, and NF-κB, leading to the transcription of target genes, and generating pro-inflammatory effector molecules [82]. Further cell stress then further activates NF-κB and downregulates EAAT2. Ultimately, this loop may potentially lead to hyperexcitability, extrasynaptic NMDA activation, and ischemia. xCT = cystine/glutamate transporter, ROS = reactive oxygen species, EAAT2 = excitatory amino acid transporter 2, TLR = Toll-like receptor, RIG-I = retinoic-acid-inducible gene I, mGluR = metabotropic glutamate receptor, IDO = indoleamine 2,3-dioxygenase, NF-κB = nuclear factor kappa-light-chain-enhancer of activated B cells. Figure was created with the BioRender software.

The consequences of reactive astrogliosis and microglial activation following SARS-CoV-2 infection can thus initiate a process of misguided LTP, excitotoxicity, and oxytosis, contributing to the development of NP symptoms and chronic fatigue observed in a subset of PCS long haulers (Figure 3). A study using a murine model of mild SARS-CoV-2 infection limited to the respiratory system, along with human brain tissue samples, showed elevation of CNS cytokines, hippocampal neurogenesis impairment, and white-matter-selective microglial reactivity [80]. While there are several studies on acute CNS injury due to SARS-CoV-2, little is known regarding the long-term neurological sequelae. A recent longitudinal study of 100 confirmed COVID-19 patients in Sweden was the first follow-up study on CNS plasma biomarkers in PCS. The study found that CNS biomarkers normalize following the acute illness, independent of disease severity or the persistence of symptoms, hinting that PCS might not be due to glial activation [81]. Considering the increasing number and the high diversity observed in PCS manifestations, further studies are urgently needed, as the authors concluded [81]—especially with recent reports showing increased frequency of NP manifestations one year post-illness compared to PCS at 6 months [9].

### 2.3. SARS-CoV-2 and the Hypothalamic–Kisspeptin Neurons

The possible routes of SARS-CoV-2 brain infection have been an issue of debate since the novel virus emerged. Due to the high percentage of patients presenting with loss of smell, the olfactory nerve has been proposed as a mechanism of viral entry to the brain via axonal transport [44]. However, there is still insufficient evidence to support this hypothesis. The majority of olfactory neurons do not express the required viral entry proteins, and infected olfactory receptor neurons in some studies lack the axonal projections needed to transport the virus into the brain [83].

**Figure 4:**
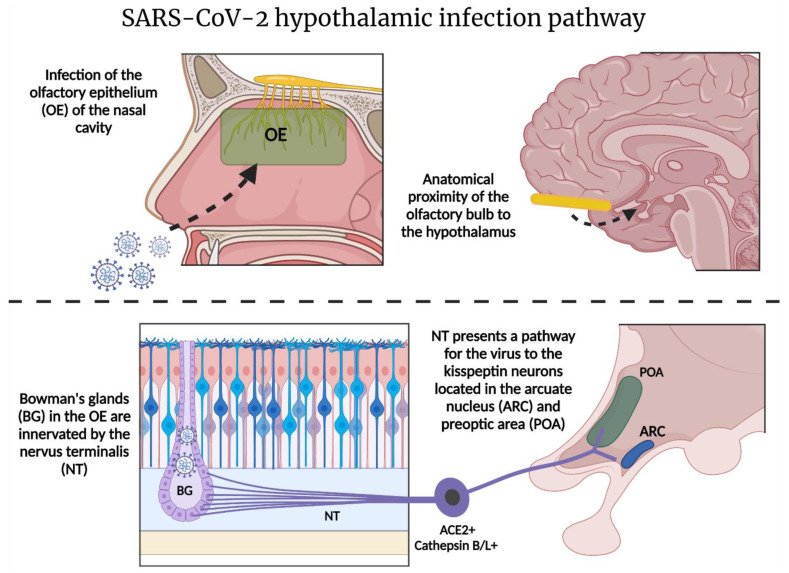
Theoretical SARS-CoV-2 hypothalamic infection pathway: SARS-CoV-2 could utilize the olfactory epithelium for host entry [83]. Anosmia is a common symptom in COVID-19, and axonal transport through the olfactory nerve has been proposed as the mechanism. Nonetheless, most olfactory sensory neurons do not express the required proteins, and lack the projections needed to reach targets in the brain [44]. The olfactory epithelium, however, expresses entry proteins necessary for SARS-CoV-2 [83]. Alternatively, the nervus terminalis (NT) may potentially provide a direct route to critical brain targets such as the hypothalamus. NT afferent fibers innervate the Bowman’s glands and express both ACE2 and cathepsins B and L [44]—the viral entry proteins for SARS-CoV-2. NT fibers terminate in the preoptic area (POA) of the hypothalamus [93], potentially providing SARS-CoV-2 with access to the hypothalamic neuronal network. Specifically, within the POA, a group of neurons referred to as the “kisspeptin neuronal network” (KNDy) might be affected. The KNDy neurons are mainly expressed in POA and the arcuate nucleus (ARC) [91], where they play a vital role in pituitary hormone secretion, thermoregulation, and autonomic response, and provide neuronal projections to critical limbic brain regions [94,95,96,97]. Figure was created with the BioRender software.

The nasal epithelial cells have been proposed to play a role in initial infection. SARS-CoV-2 entry factors are highly expressed in nasal epithelial cells [84]. Interestingly, the terminal nerve or “nervus terminalis”—the 13th cranial nerve, described as cranial nerve “zero”—has fibers that ascend from the olfactory epithelium and Bowman’s glands to important limbic areas such as the hypothalamus [85,86], which may serve as a hub for viral brain infections [87]. Recently, the nervus terminalis has been demonstrated to express both ACE2 and cathepsins B and L needed for SARS-CoV-2 entry, thereby providing the virus with an alternative route directly to targets in the brain [45] (Figure 4). In the study, the majority of nervus terminalis neurons that expressed the viral entry points were gonadotropin-releasing hormone (GnRH)-positive neurons that are thought to have neurosecretory functions.

The function of the nervus terminalis is not yet fully understood. During embryonic development, this nerve plays an important role in luteinizing hormone-releasing hormone (LHRH)/GnRH neurons’ development and migration to preoptic hypothalamic areas [88,89]. Failure of migration can lead to primary hypogonadism, and is associated with Kallmann syndrome, suggesting a role for the cranial nerve in the hypothalamic–pituitary–gonadal (HPG) axis [90,91]. Therefore, the nervus terminalis has been regarded to play a role in autonomic responses through GnRH via the kisspeptin–hypothalamic network [85]. The kisspeptin (Kiss1)-expressing neurons are two populations of glutamate-coexpressing neurons in the hypothalamus, which are mainly located in the arcuate nucleus (ARC) and in the preoptic area (POA) [92]. This connection gives SARS-CoV-2 a path to alter reproductive and autonomic responses, while the fact that Kiss1 neurons are also partly glutamatergic makes their synapses vulnerable to the pathological processes outlined above.

Kiss1 is a ligand for the G-protein-coupled receptor 54 (GPR54). Loss of function of GPR54 causes hypogonadotropic hypogonadism [98]. Recent discoveries in the kisspeptin–hypothalamic network have shown that kisspeptin plays a central role in human reproductive health. Kisspeptin neurons (KNDy) co-express neurokinin B (NKB) and dynorphins, and are key regulators of GnRH secretion [94,99]. Nevertheless, only kisspeptin can activate GnRH neurons to drive episodic hormone secretion [100]. Kisspeptin stimulation can drive the mechanism behind GnRH pulsatile secretion. GnRH regulates LH and follicle-stimulating hormone (FSH) synthesis and release from the pituitary gland which, subsequently, stimulates ovulation, follicle development, and the secretion of gonadal hormones. On the other hand, ovarian gonadal hormones provide feedback to the hypothalamus and pituitary gland. For example, the negative feedback by estrogen’s suppression of gonadotropin secretion is mediated by KNDy neurons [101]. In addition, estrogen’s increase during the follicular phase provides the positive feedback to stimulate the release of gonadotropins through its actions on Kiss1 neurons [102,103]. In addition to their role in reproductive health, kisspeptin neurons play a role in the hypothalamic circadian network. Kiss1^ARC^-silenced mice exhibit decreased home-cage and running-wheel activity [95], which may be indicative of increased fatigue and/or depression-like and anxiety-like behaviors [95,104].

With regards to the hypothalamic–kisspeptin network in PCS, hypogonadism and altered gonadotropin hormones levels have been observed in COVID-19 [105,106]. However, whether HPG abnormalities and/or a direct effect on the gonads is the cause remains a matter for debate [107,108]. Additionally, the hypothalamic–pituitary–adrenal (HPA) axis has been implicated in COVID-19 illness [109]. Interestingly, as the elimination of estrogen signaling on Kiss1 neurons leads to ovulatory failure [110], menopause has been described as an independent risk factor for female COVID-19 patients [111]. Moreover, many PCS manifestations, although more drastic, seem to overlap with menopausal transition. Heat intolerance, irregular cycles, and abnormal autonomic responses have been reported in PCS [18,112,113]. During the perimenopausal period, loss of negative feedback from sex hormones leads to alterations in the hypothalamus and hypertrophy of KNDy neurons, as their signaling is unopposed. KNDy neurons are altered in postmenopausal women, and contribute to hot flushes by influencing thermoregulation in the POA and facilitating cutaneous vasodilation [96,97]. These changes, coupled with an increase in NKB secretion [114] in the preoptic area, are a primary cause of postmenopausal vasomotor symptoms. Indeed, neurokinin receptor (NK3R) antagonism alleviates hot flushes and improves quality of life [115]. If KNDy neurons play a role in a subset of PCS symptoms, NK3R antagonism might be a possible treatment for a component of PCS manifestations.

**Figure 5:**
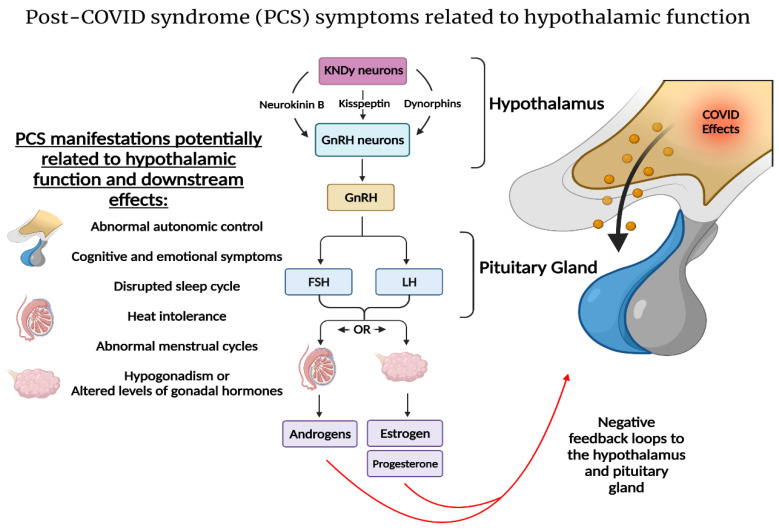
The hypothalamic circuit as a hub for PCS development: A plethora of PCS symptoms might be due to a disruption of key hypothalamic functions [117,120]. The KNDy hypothalamic neurons in the POA and ARC hypothalamic regions are vital for maintaining thermoregulation and reproductive health [93]. KNDy neurons are key regulators of GnRH release [99,100] and HPG axis homeostasis. COVID-19 illness due to viral invasion and/or the subsequent hyperinflammatory state may alter KNDy neurons and contribute to the heat intolerance, altered gonadal hormones, and abnormal menstrual cycle noted in PCS [18,104,106]. Furthermore, the paraventricular nucleus (PVN) of the hypothalamus plays a vital role in maintaining autonomic homeostasis and neurocognitive functions [118,120], which might be compromised in PCS, and might explain the neurological sequelae in PCS [8,9,24]. Figure was created with the BioRender software.

As already discussed, in addition to a direct olfactory route for SARS-CoV-2 to the hypothalamus, leakage of pro-inflammatory cytokines into the CNS can contribute to neurological manifestations. Upon reaching the hypothalamus, this can lead to the autonomic dysfunction, dysregulation of sleep cycles, and cognitive dysfunction observed in PCS [116]. Likewise, autonomic dysfunction has been observed in PCS [117,118], and is more exacerbated in those with neurological symptoms [117]. The hypothalamic paraventricular nucleus (PVN) is considered to be a key factor in autonomic function, integrating the multiple sources of afferent input to generate an integrated autonomic output [119]. In addition, patients with autonomic dysfunction show disrupted hypothalamus connectivity [120]. The symptoms of PCS can thus be explained by a defective PVN acting as a hub for the activation of microglia and astrocytes. The dysfunctional hypothalamus can thus contribute to the emotional, cognitive, thermoregulatory, and HPA/HPG abnormalities in PCS patients [121] (Figure 5).

## 3. Conclusions

Post-COVID-19 syndrome shows high prevalence and multisystem involvement among survivors. The syndrome overlaps with ME/CFS and previous post-SARS syndromes. Nonetheless, a lack of consensus on the characterization of PCS needs to be addressed. The syndrome is likely to involve many immunological processes. Manifestations of PCS may be partially explained by a neuroinflammatory process involving the activation of astrocytes and microglia. The hypothalamus and its network are implicated in the ongoing neuroinflammation and might be a major cause of some of the persistent symptoms. As vaccine rollout increases and the world is looking into a post-COVID era, developing accurate laboratory and clinical guidelines for PCS is crucial to define and identify PCS. Long-term specialized brain imaging and glial marker studies are urgently needed to determine whether PCS exhibits a specific imprint in the CNS. There are several clinical trials on various anti-inflammatory agents—such as corticosteroids—currently being performed, and these studies may help to improve our understanding—especially if they are aided by extensive measurements of the relevant biomarkers.

## Figures and Tables

**Table 1:** Summary of the described manifestations of PCS; (*) represents the most commonly reported symptoms [4,8,18,20,21,24].

Respiratory sequelae	Dyspnea *, cough, sore throat, altered diffusion capacity, restrictive pattern, obstructive pattern
Cardiac sequelae	Palpitations, chest pain, myocarditis
Gastrointestinal sequelae	Vomiting/nausea, diarrhea
Neurological sequelae	Anosmia, loss of taste, anxiety *, depression, sleeping difficulties, concentration/memory problems *, dizziness, chronic fatigue*, headache
Other sequelae	Joint pain, post-exertional malaise *, increased incidence of pain, antihypertensive, and antidepressant drugs

## Data Availability

Data for this review were identified by searches of MEDLINE, Google Scholar, and PubMed, as well as references from relevant articles using the search terms “Post-COVID” OR “Post-SARS” OR “SARS-CoV-2” AND “astrocytes activation” OR “microglia activation” OR “Kisspeptin neurons” OR “hypothalamus”. Only articles published in English between 2000 and 2021 were considered.
